# CONTACT URTICARIA: PRESENT SCENARIO

**DOI:** 10.4103/0019-5154.55639

**Published:** 2009

**Authors:** Ruchi Bhatia, Ali Alikhan, Howard I Maibach

**Affiliations:** *From the Department of Dermatology, The Northeastern Ohio Universities College of Medicine, Rootstown, Ohio*; 1 *From the Department of Dermatology, University of California Davis, School of Medicine, Sacramento, California*; 2 *From the Department of Dermatology, University of California, San Francisco School of Medicine, San Francisco, California USA.*

**Keywords:** *Contact urticaria*, *contact dermatitis*, *contact urticaria syndrome*, *urticaria*

## Abstract

Immunological contact urticaria is a hypersensitivity reaction that appears on the skin following contact with an eliciting substance. Recent advances in our understanding of the molecular mechanism and pathogenesis of this reaction have altered its classification, diagnosis, and treatment. We discuss classification, epidemiology, diagnosis, testing, and treatment options that are available to patients with contact urticaria.

## Introduction

Contact urticaria (CU), first described by Fischer in 1973, is defined as the development of a wheal-and-flare reaction at a site where an external agent contacts the skin or mucosa.[1,2] Symptoms of contact urticaria range from pruritic, localized wheal-and-flare reactions to generalized urticaria and anaphylaxis. The wheal-and-flare response appears as a raised, burning, and/or pruritic swelling of the skin, often accompanied by angioedema.[3] Diagnosis is made primarily on the basis of history and clinical presentation, without extensive laboratory investigation. Over the last 30 years, we have seen an increasing recognition of contact urticaria.[4,5]

Contact urticaria is divided into two subtypes: Immunological CU and nonimmunological CU. Nonimmunological CU is an immediate reaction not requiring prior exposure to the substance, while immunological CU is a Type 1 IgE-mediated hypersensitivity reaction in which the patient's immune system has been previously sensitized to the eliciting substance.[6] Previous sensitization can occur through contact with or exposure to the skin, mucous membranes, respiratory tract, or gastrointestinal tract.

Initial presentation of the reaction appears within minutes to hours of exposure, affecting normal or eczematous skin with nonspecific symptoms.[3] Local sensations may occur without visible changes to the skin. Symptoms usually remain in the contact area, in the setting of nonimmunological CU, while other organs may be involved following immunological CU, leading to conjunctivitis, rhinitis, asthmatic attack, or even anaphylactic shock.[3] Maibach and Johnson defined Contact Urticaria Syndrome (CUS) as a group of inflammatory reactions of the skin, occurring within minutes after contact with the eliciting substance.[7] They further described the four stages of progression in CUS [Table 1].[6]

**Table 1:** Stages of progression in contact urticaria[6]

Stage	Description
1	Localized reaction (redness and swelling) with nonspecific symptoms (burning, itching, tingling)
2	Generalized reaction
3	Extracutaneous symptoms (rhinoconjunctivitis, orolaryngeal and gastrointestinal dysfuntion)
4	Anaphylactic shock

## Pathophysiology

Nonimmunological contact urticaria is the most common type of CU, occurring without prior exposure to an eliciting substance as a direct result of urticants on blood vessels.[2] Symptoms usually develop within hours, remain up to 24 hours, and vary depending on the substance and site of exposure. This type of urticaria generally does not go beyond the localized reaction described in Stage 1 [Table 1], presenting with redness and swelling along with nonspecific symptoms of burning and itching.

Due to the lack of response to antihistamines and the positive response to acetylsalicylic acid (ASA) and non-steroidal anti-inflammatory drugs (NSAIDS) in nonimmunological urticaria, it has been proposed that the pathophysiology involves prostaglandin release from the epidermis rather than histamine release from the mast cells, as previously assumed.[8] In testing the previously assumed release of histamine in nonimmunological urticaria, hydroxyzine and terfenadine, both H1 antihistamines, were unsuccessful in inhibiting reactions to benzoic acid, cinnamic acid, cinnamic aldehyde (cosmetics), methyl nicotinate, and dimethyl sulfoxide, suggesting that histamine is not the main mediator of nonimmunological CU.[9] ASA works as an anti-pyretic and anti-inflammatory agent, to inhibit urticarial symptoms for up to four days.[9]

The release of histamine is the major mechanism of action seen in immunological urticaria. The pathophysiology of immunological CU differs from nonimmunological CU, as it occurs due to the production of allergen-specific IgE following prior sensitization to a causative agent.[10] After the initial binding of allergen-bound IgE to mast cells, basophils, Langerhans cells, and eosinophils, histamine (as well as leukotrienes and prostaglandins) are released.[11,12] These inflammatory mediators lead to mucus secretion, airway smooth muscle contraction, and mucosal edema in the early- and late-phase allergic response.[9] Stimulation of FceRIs, high affinity IgE receptors on mast cells, further leads to upregulation, synthesis, and secretion of more inflammatory agents.[9,13] The histamine-mediated reaction of immunological CU may pass the localized stage 1 reaction and progress to generalized, extracutaneous, and anaphylaxis reactions [Table 1].

## Diagnosis

Many causes of immunological CU have been described, thus emphasizing the need for a thorough history as an initial step toward diagnosis. An individual presenting with urticaria should be questioned about: time and duration of onset, presence of nonspecific symptoms, wheal and angioedema distribution, family history, drug use, previous allergies and history of previous anaphylaxis, reaction to insect stings, stress, recent travel, vocation, hobbies, relationship to menstrual cycle, and effect of the urticaria on the quality of life.[14] A study of 112 patients, assessing the effect of dermatological disorders on the quality of life, found that patients with allergic contact dermatitis and urticaria displayed the most extensive disturbances in physical and psychosocial functioning.[15] These patients displayed the highest levels of somatic symptoms, anxiety, insomnia, and symptoms of depression. Urticaria, itself, displayed the highest level of tension, hostility, and fatigue among patients.

Eliciting factors for CU typically arise from occupational settings. A 12-year retrospective study in Australia reported that of the 151 patients diagnosed with CU, 143 (94.7%) were work-related.[5] The most frequently affected occupations were healthcare workers, food handlers, and hairdressers with the most commonly affected sites of the body being the hands (125 patients out of 143), arms (35 of 143), and face (30 of 143). The most common culprit was natural rubber latex (75 out of the 143 cases due to latex) and most patients were female (90 of 143). A Finnish occupational CU study demonstrated that bakers, preparers of processed food, and dental assistants were at highest risk of developing occupational CU.[16] Additional occupations at high risk for developing CU included agricultural and dairy workers, electronics workers, hairdressers, and medical and veterinary workers.[5,17] CU has also been described in those exposed to cyclic acid anhydrides (i.e., welders, painters, plumbers, chimneysweeps, packers, electricians),[16] produce handlers,[18] gardeners (with exposure to common ivy, rosemary, basil, sage, oregano, marjoram, thyme, and peppermint, among other plants),[19] and those using lip plumpers.[20]

The most common cause of immunological CU by a single agent is said to be the presence of latex proteins in rubber agents such as gloves, condoms, and balloons.[12,21] Thus, occupations with the maximum exposure to latex products are more likely to develop immunological CU, including healthcare or dental workers.[19,22] The prevalence of latex reaction has been estimated to be 0.7% in the general population, while in healthcare workers it has been noted to be up to 17%.[23] Cross reactivity, sensitization to one protein resulting in a reaction to a similar (but different) protein, is often seen with certain foods such as avocado, chestnuts, and bananas in those with latex-induced immunological CU.[3] This mechanism allows two structures with similar chemical reactivity and molecular shape to activate the same receptors.[24]

After a thorough history is taken, the physician should follow with a focused physical examination, ensuring that antihistamines have not been used within two days of performing the examination.[3] Testing commonly employs a step-wise approach and may include the open test, prick test, scratch test, and use test, making sure to include positive and negative controls during each step.[3] Saline is used as a negative control in these tests. The open test is first performed on nonaffected skin and if negative, on slightly affected or previously affected skin.[3,25] The open test is performed by using 0.1 mL of the suspected urticarial substance and spreading it over a 3 × 3 cm area. The reaction is measured at 20, 40, and 60 minutes. Three to four readings must be taken in an hour, to avoid missing the positive reactions. This includes follicular erythema followed by a wheal or wheal-and-flare response. The ventral forearm is used for immediate responses, while the back is used for delayed hypersensitivity reactions. Immunological CU can usually be seen within 15 to 20 minutes, while nonimmunological CU can be delayed up to one hour. When performing an open test, physicians should take precautions against anaphylaxis.

If the open testing is negative, occlusive patch testing may be performed. However, the prick test is usually performed next in the diagnosis of CU, and is considered the diagnostic method of choice if open testing is negative. The test substance is applied to the volar aspect of the forearm and pierced into the skin using a lancet. As only a small amount of allergen is introduced into the skin, there is a low risk of anaphylaxis. Reading of a prick test is usually performed in 30 minutes. Although not used as often, the scratch test is more useful for non-standardized allergens. The area of the skin is scratched with needles after the allergen has been applied. Similarly, this test is also read in 30minutes after contact with the allergen. Finally, the use test requires the patient to use the causative agent that resulted in contact allergy. For example, a person with latex-induced CU would wear latex gloves during testing.

## Treatment

Emphasis for treatment of CU should be placed on prevention–thorough history taking and appropriate clinical testing will help determine the problematic materials. Once the diagnosis of contact urticaria is made, avoidance is almost always possible. In case of contact, patients should carry antihistamines and receive education on self-administration of subcutaneous epinephrine.[26]

Because contact with an eliciting substance may still occur, management is divided into first-, second-, and third-line therapies [Table 2]. In addition to patient education, first-line therapy includes the use of H1 receptor antihistamines. The ability of these agents to suppress the histamine-induced wheal-and-flare response has been well studied in urticaria they may decrease both the number and the duration of wheals.[27] H2 receptor antagonists, including cimetidine, ranitidine, nizatidine, and famotidine, are often used along with H1 receptor antagonists, because 15% of the skin receptors are of the H2 type.[24] The improved tolerability of newer, nonsedating antihistamines may give them a clear advantage over older antihistamines and corticosteroids.[28]

**Table 2:** First, second, and third-line therapies for contact urticaria

Therapy	Class	Adverse events
First line	H1 receptor antagonist	Sedating and strong anticholinergic
	H2 receptor antagonist	Sedating
	Second generation antihistamine	Less sedating (act on other mediators)
Second line	UV radiation/photochemotherapy	Burns, DNA and collagen damage, cataracts, aging
	Tricyclic antidepressants	Highly sedating and strong anticholinergic
	Corticosteroids	Osteoporosis, ulcers
	Leukotriene receptor antagonists	Hypersensitivity, GI disturbances, bleeding
Third Line	Immunomodulatory agents (Cyclosporine)	Renal toxicity with long-term use
	Immunomodulatory agents (Methotrexate)	Hepatotoxicity with long-term use

These agents should infrequently be needed, as preventing further exposure remains the ideal approach.

Slater *et al.,* described the increased effectiveness of second-generation drugs cetirizine, mizolastine, and ebastine over certain first-generation agents, in the treatment of generalized urticaria.[29] In addition to their antagonistic effects on H1 receptors, second-generation antihistamines also act on other allergic mediators, such as leukotrienes and prostaglandins; they also produce fewer CNS side effects. A study comparing the duration and degree of suppression of the histamine-induced, wheal-and-flare reaction demonstrated that cetirizine displayed the longest duration of inhibition, followed by loratadine and chlorpheniramine.[30]

Second-line therapies may be employed if a trial of antihistamines fails. UV radiation and photochemotherapy have been used successfully in chronic urticaria, and may be effective in CU.[31,32] In one study, an inhibitory response of two weeks in nonimmunological urticaria was seen with both UVA irradiation (long-wave radiation above 340nm) and UVB irradiation (long-wave radiation above 300 nm).[6] UVA and UVB radiation may suppress flares by inducing T-lymphocyte apoptosis and promoting reductions in mast cells and Langerhans cells in the dermis. Radiation also reduces pruritis by inhibiting the release of histamine from mast cells and basophils. Adverse effects include erythema, hyperpigmentation, polymorphic light eruption, and pruritis (due to radiation-induced dryness); chronic side effects include photoaging and skin cancer.

Tricyclic antidepressants, such as doxepin, often have potent H1 and H2 receptor antagonist activity.[31] Doxepin may be more effective than diphenhydramine in the control of urticaria, but it is highly sedating and has strong anticholinergic side effects. A gradual increase in dosage (over time) at bedtime may reduce the side effects, and could be useful in conjunction with H1 antihistamine therapy.[33] Systemic corticosteroids may be used when rapid and complete disease control is needed. In a non-blinded study of 109 patients, both corticosteroids and antihistamines were effective in controlling urticarial reactions, but symptoms ceased earlier in corticosteroid-treated patients.[34] However, corticosteroids are not prescribed for long-term therapy, as they may cause possible hyperglycemia, osteoporosis, peptic ulcers, and hypertension. If prolonged corticosteroid use is essential, corticosteroid-sparing immunosuppressive modalities are added to the treatment regimen.[35] Leukotriene receptor antagonists, including montelukast, zafirlukast, and zileuton, have also proven to be effective by inhibiting potent inflammatory mediators of inflammation.[12,36]

The third-line therapy for urticaria involves the use of immunomodulatory agents such as cyclosporine and methotrexate. Long-term therapy is limited in these agents due to adverse side effects, including hypertension and renal toxicity.[35] Kessel and Toubi described 100 patients with severe urticaria whom they treated with 2-3 mg/kg cyclosporine; 40% of the patients displayed disappearance of urticarial symptoms, while 30% displayed significant improvement.[37] Caution should be taken to avoid significant side effects such as hypertension, peripheral neuropathy, and increased serum creatinine levels, by decreasing the initial dosage.

We emphasize that prevention (of further exposure) remains the desideratum of therapy. When extracutaneous organs are involved, this is especially important. Taken together, the diagnosis and prophylaxis should greatly lessen morbidity. A high priority research goal is to develop a reliable screening assay for new immunological contact urticaria.
